# What the Controlled-Vocabulary Layer Cannot Say About Where a Depression Biomarker Was Measured: A Meta Research Analysis of MeSH Sampling-Compartment Coding, January 2024–June 2026

**DOI:** 10.62641/aep.2463

**Published:** 2026-10-15

**Authors:** Walter Paganin

**Affiliations:** ^1^Department of Systems Medicine, University of Rome Tor Vergata, 00133 Rome, Italy

**Keywords:** Biomarkers, Depressive Disorder, Major, Bibliometrics, Databases, Bibliographic, Medical Subject Headings

## Abstract

**Background:**

Peripheral biomarkers for major depressive disorder are accessible but correspond poorly to central nervous system processes, a point the evidence base can address only if its record registers the compartment in which each marker was measured. To determine, by meta research rather than evidence synthesis, whether the indexed record of recent depression biomarker research registers the peripheral-central distinction, separating omission by indexers from absence mandated by the controlled vocabulary. That peripheral markers are imperfect proxies for central processes is established prior knowledge; the question here concerns the bibliographic infrastructure through which that literature is retrieved.

**Methods:**

PubMed records published between 1 January 2024 and 30 June 2026 matching a title/abstract query for depression and biomarker terms were retrieved (Tier 1, n = 1166); the MEDLINE-indexed subset formed the analysable corpus (Tier 2, n = 825). We extracted marker-level descriptors and site qualifiers, determined from the 2026 MeSH descriptor file whether /blood and /cerebrospinal fluid are allowable per descriptor, and stratified by publication type.

**Results:**

Of 1166 retrieved records, 341 (29.2%) carried no MeSH descriptor; in the closed year 2024, 70 of 317 (22.1%) remained unindexed, so roughly one article in five is permanently absent from the structured record. Within the 825 indexed records, 494 (59.9%) carried no marker-level descriptor and 658 (79.8%) no site qualifier. Of 1211 distinct descriptors, 681 (56.2%) do not permit /blood at all, most of them terms for which a sampling compartment would carry no meaning; restricted to the 243 marker-level and class-level descriptors, 30 (12.3%) are barred, C-reactive protein among them, the most frequent marker descriptor in the corpus (26 records), though the same descriptors can receive /cerebrospinal fluid. Primary studies recorded a compartment three times more often than reviews (38.8% vs 14.8%; χ^2^ = 52.1, *p* < 0.001), but recording tracked qualifier eligibility more closely than study design, rising from 5.0% to 59.4% with the number of /blood-eligible descriptors a record carried.

**Conclusions:**

The controlled-vocabulary layer cannot reliably recover the sampling compartment of depression biomarker research, for two structural reasons: the peripheral compartment is unrecordable for an entire marker class, and recording depends on how many of a record's descriptors the vocabulary permits to carry the qualifier at all. A distinction that cannot be recorded cannot be aggregated, audited or contested. The inflammatory endotype literature of difficult-to-treat depression is thereby constrained by a blind spot at the level of retrieval rather than biology.

## Introduction

Biomarkers in major depressive disorder (MDD) are pursued in order to refine diagnosis, stratify phenotypes and predict treatment response across immuno-inflammatory, neuroendocrine, neurotrophic and neuroaxonal/glial domains [1, 2]. The clinical motivation is concrete rather than theoretical: inflammatory signalling has become an explicit therapeutic target in depression [3], and data-driven attempts to resolve the disorder into biological subtypes depend on being able to identify, retrieve and pool the relevant marker studies from the published record [4]. The immuno-inflammatory case is the strongest: meta-analytic data in 5166 patients and 5083 controls confirm elevated peripheral C-reactive protein (CRP), interleukin-6 (IL-6) and tumour necrosis factor-α (TNF-α) in depression and, more informatively, greater inter-individual variability among patients than among controls [5, 6]. Increased variance is the signature of a mixture rather than of a uniform shift, and hence of an inflamed subgroup that stratification might recover [7]. A scoping review of the recent literature reached a convergent conclusion from the opposite direction: the group-level associations are reproducible, but their translational value is constrained by methodological heterogeneity and by the limited neural specificity of peripheral measures [8]. Systematic appraisals of clinical utility concur that no biomarker is yet ready for diagnostic use [9]. Almost all of this evidence is peripheral. Blood, serum, plasma, saliva and urine are accessible, inexpensive and scalable; cerebrospinal fluid (CSF) is none of these. The inference the field consequently requires, from a peripheral measurement to a statement about the brain, is not, however, licensed by feasibility. It has been examined directly, and the answer was discouraging. A systematic review and meta-analysis identified only 29 studies with paired blood–CSF inflammatory measurements across the whole of psychiatry and neurology and across decades, of which 21 were meta-analysable; the pooled blood–CSF correlation was low (r = 0.21), significance was confined to IL-6 (r = 0.26) and TNF-α (r = 0.30) after exclusion of outliers, and correlations rose only in participants above 50 years of age (r = 0.46) and in autoimmune disease (r = 0.35). Peripheral concentrations cannot be assumed to mirror central activity, and the coupling that does exist appears conditional on blood–brain barrier state rather than being a stable property of any analyte [10].

The aim of this meta research study was to determine whether the indexed record of recent depression biomarker research registers the compartment in which each marker was measured, and, where it does not, whether the omission reflects indexing practice or is mandated by the controlled vocabulary itself. The stake is not academic. Anti-inflammatory augmentation, immunomodulatory trials and inflammation-based stratification of difficult-to-treat depression (DTD) all presuppose that a peripheral index identifies patients in whom a central inflammatory process is operating. Should that presupposition fail, trials will continue to be enriched on the wrong variable, and negative results will continue to be attributed to the intervention rather than to the selection criterion. The mechanistic rationale is not itself in doubt: inflammatory signalling is coupled to the reward and effort–cost circuitry that underpins anhedonia and motivational deficit, most visibly in treatment-resistant illness [3, 11, 12]. What is in doubt is whether a peripheral measurement can identify the patients in whom that circuitry is affected. A prior question, however, precedes that one and has not to our knowledge been asked. A literature can be compelled to confront a validation gap only if its record-keeping registers the variable on which the gap depends. Before asking what the field has done with the blood-to-brain problem, one must ask whether the field’s own record, the indexed metadata through which its output is stored, retrieved and cumulated, is even capable of registering the distinction on which that problem turns. If the sampling compartment is not a retrievable element of the record, then the question cannot be posed to the literature at all, no matter how it is searched. The distinction bears directly on what is claimed here. That peripheral inflammatory markers are imperfect proxies for central processes is established, repeatedly demonstrated, and is not the finding of this study. What is reported here lies one level beneath the biology: the structured record through which this literature is stored and retrieved cannot express the variable on which that established limitation turns. The consequence is not that the field holds a mistaken belief about its markers, but that it cannot systematically interrogate the belief it holds, because the studies bearing on it cannot be assembled on the feature that distinguishes them. Throughout, “the indexed record” denotes the MeSH controlled-vocabulary layer of the MEDLINE record, the descriptor and subheading fields through which the literature is filtered and pooled. It does not denote the whole of a PubMed record, and claims below are bounded accordingly. We map two and a half years of the depression biomarker literature at the level of its MeSH metadata, asking: (i) what proportion of the retrieved literature is indexed at all, and how much of the shortfall is permanent rather than transient; (ii) among indexed records, what proportion carry a marker-level descriptor; (iii) whether the sampling compartment is recorded, and at what granularity; (iv) whether the peripheral qualifier was an allowable option under MeSH rules, so that genuine omission can be separated from vocabulary-mandated absence; and (v) whether compartment recording depends on study design.

## Materials and Methods

### Study Design

This is a meta research study, research whose object is the conduct and reporting of research rather than a clinical or biological question, and specifically a bibliometric analysis of indexing metadata. It is neither a systematic nor a scoping review: no eligibility criteria were applied to individual records, none was excluded on the basis of its findings, no data were extracted from full texts, and no risk-of-bias or effect-size assessment was undertaken. PRISMA and PRISMA-ScR reporting is accordingly neither applicable nor claimed; Fig. 1 documents the construction of the corpus, not a screening cascade, and records without a marker descriptor constitute a study outcome rather than an exclusion. Outcomes are descriptive frequencies, with one exception specified below. This design supports claims about the structure, completeness and retrievability of the evidence base. It cannot support claims about the magnitude or validity of any biomarker association; where such claims appear below, they are sourced to external literature and identified as such.

**Fig. 1.**
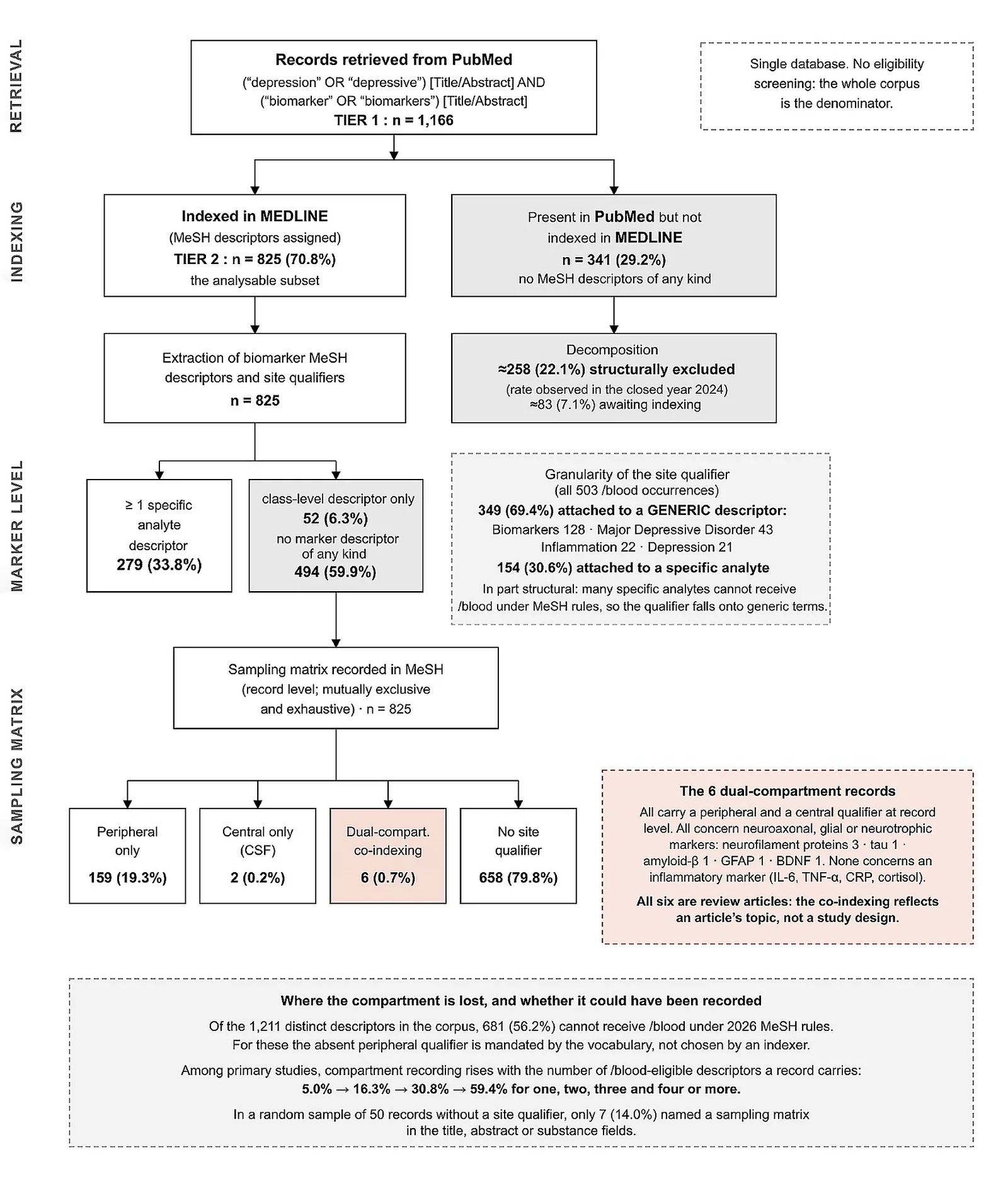
**Construction of the corpus and of the analysable subset**. Notes. Tier 1 comprises all retrieved articles describing biomarker research in depression; Tier 2 is the MEDLINE-indexed subset used for all MeSH-based analyses, which are therefore unaffected by indexing lag. The unindexed fraction is decomposed using the rate observed in the fully matured 2024 publication year. This is a descriptive flow diagram, not a PRISMA flow diagram: records without marker descriptors are a study outcome, not an exclusion. Marker-level and sampling-matrix categories are each mutually exclusive and sum to 825. Dual-compartment co-indexing records the co-occurrence of a peripheral and a central qualifier within a record, not a paired sampling design. BDNF, brain-derived neurotrophic factor; CSF, cerebrospinal fluid; GFAP, glial fibrillary acidic protein.

### Corpus Construction: A Two-Tier Denominator

Records published between 01/01/2024 and 30/06/2026 were retrieved from PubMed in July 2026 with a title/abstract text-word query, (“depression”[Title/Abstract] OR “depressive”[Title/Abstract]) AND (“biomarker”[Title/Abstract] OR “biomarkers”[Title/Abstract]), restricted by publication type to Clinical Trial, Randomized Controlled Trial, Meta-Analysis and Review, together with the PubMed systematic review subset (systematic[sb]). This returned 1166 records (Tier 1), the population of empirical and synthesising articles that describe themselves as biomarker research in depression. The publication-type restriction confines the corpus to article types that report or synthesise empirical findings, excluding editorials, letters, comments and case reports, which carry no sampling design and for which a compartment qualifier would have no referent. Retrieval was deliberately performed at the text-word rather than the MeSH level: a MeSH-restricted query would have pre-selected records already carrying biomarker descriptors and would thereby have rendered the primary outcome, the proportion of self-described biomarker articles that carry such a descriptor, circular and uninterpretable. MeSH descriptors exist only for records indexed in MEDLINE. Adding the filter AND medline[sb] returned 825 records (Tier 2), the subset on which any MeSH-based analysis is possible. All metadata outcomes reported below are computed within Tier 2, which is by construction fully indexed and therefore unaffected by indexing lag. The Tier 1 to Tier 2 shortfall is itself reported as a finding. PubMed applies indexing retroactively and continues to acquire records, so counts are not exactly reproducible at a distance; those reported throughout are those of the extraction date.

A corollary of the retrieval strategy must be stated at the outset, because it bears on the interpretation of every downstream frequency: the corpus is dominated by review articles.

Two features converge on this composition. A title/abstract query for the co-occurring terms “depression” and “biomarker” preferentially retrieves synthesising and discovery-oriented articles, which name both concepts explicitly, over primary wet-lab reports, which frequently name a specific analyte instead; and the publication-type filter admits three synthesising categories against two primary ones. The composition is therefore a property of the retrieval strategy rather than a defect of the corpus, and publication type is carried forward as an explicit stratifying variable.

### Separating Indexing Lag From Structural Exclusion

National Library of Medicine indexing lags publication, so a corpus extending to June 2026 necessarily contains recent records not yet processed. To distinguish transient lag from permanent exclusion, the same queries were re-run restricted to the closed publication year 2024, for which indexing has had more than eighteen months to complete. Records still unindexed in that window cannot be awaiting indexing and are, on any reasonable reading, permanently outside MEDLINE.

### Extraction and Operational Definitions

The 825 Tier 2 records were exported in MEDLINE format and parsed with a purpose-written script (**Supplementary Material S4**), which split each MH field into its descriptor and subheadings. A marker-level descriptor was defined as a MeSH descriptor naming a specific, measurable molecular analyte (protein, peptide, hormone, metabolite, lipid or nucleic-acid species) or a defined cell count. Excluded were the category term Biomarkers, disease and syndrome descriptors, anatomical structures, specimen types, process terms such as Inflammation, and broad chemical-class terms that do not identify a specific analyte (Cytokines, MicroRNAs, Neurotransmitter Agents, Inflammation Mediators, Antioxidants, Exosomes, Extracellular Vesicles), the last reported separately as class-level descriptors. Included and excluded descriptors, the latter with the reason for each exclusion, are all listed in **Supplementary Table S1**, so the definition can be audited.

Sampling matrix was coded from the subheading attached to a descriptor. Records, and separately descriptors, were classified as peripheral only (/blood, /urine), dual-compartment (peripheral plus central), central only (/cerebrospinal fluid), or site not specified, four mutually exclusive and exhaustive categories summing to the record count. Subheadings such as /analysis and /metabolism carry no anatomical information and counted as “no site qualifier”. The dual-compartment class is defined at the record level: a record enters it when any descriptor carries a peripheral qualifier and any descriptor in the same record carries the central qualifier, whether or not on the same descriptor. Because this aggregation is a property of the record rather than of a study design, the class is labelled “dual-compartment co-indexing” rather than “paired sampling”: it registers that an article’s metadata reference both compartments, not that paired specimens were collected.

Allowable-qualifier eligibility. A subheading can be assigned only if it is an allowable qualifier for that descriptor, and the National Library of Medicine (NLM) defines allowable qualifiers by descriptor category, disallowing /blood for several categories entirely. An absent /blood qualifier is therefore ambiguous: it may reflect genuine omission, where the qualifier was allowable but not assigned, or mandated absence, where it could never have been assigned. To separate the two, the 2026 MeSH descriptor file (desc2026.xml) was downloaded from the NLM and parsed to determine, for each of the 1211 distinct descriptors in the corpus, whether /blood and /cerebrospinal fluid were allowable qualifiers. Each descriptor was flagged accordingly, and compartment-recording frequencies were recomputed within the eligible subset alone, excluding descriptors for which the qualifier was never an option. The parsing script and the full eligibility table are provided as **Supplementary Material S4** (mesh_eligibility.py) and **Supplementary Material S2** (eligibility_table.tsv).

Publication-type stratification. Because reviews predominate and a review discusses rather than performs sampling, each record was classified as a primary study or a review from its MeSH Publication Type field, those carrying Review, Systematic Review or Meta-Analysis forming the review stratum. The sampling-matrix distribution was tabulated in each stratum, and the association between publication type and the presence of any compartment qualifier was tested with an uncorrected Pearson chi2 test on the 2 × 2 table (primary vs review × compartment recorded vs absent). Because a stratified difference alone cannot establish which variable dominates, competing determinants were then compared in logistic models of compartment recording, entering publication type, marker level, indexing depth (number of MeSH descriptors per record) and the number of /blood-eligible descriptors per record; associations between analyte class and compartment recording were tested with Fisher exact tests. These models are exploratory and were specified after the descriptive analysis. All other outputs are descriptive frequencies.

Predefined outcomes were the indexing yield at each tier; the proportion of indexed records carrying a marker-level descriptor; the distribution of sampling matrices; the granularity at which the site qualifier is attached; the proportion of descriptors for which /blood is allowable; and the dependence of compartment recording on publication type.

## Results

### Indexing Yield: One Article in Five Is Permanently Outside the Structured Record

Of the 1166 records retrieved (Tier 1), 825 (70.8%) were indexed in MEDLINE and 341 (29.2%) carried no MeSH descriptor of any kind (Fig. 1). Restricting to the closed publication year 2024, 70 of 317 records (22.1%) remained unindexed; applying that structural rate to the whole window, approximately 258 records are permanently outside MEDLINE and approximately 83 (7.1%) are awaiting indexing. The structural fraction is not a processing backlog but a standing property of the database: roughly one such article in five will never acquire a single MeSH descriptor, and is therefore unreachable by any MeSH-based search, however constructed.

### Marker-Level Descriptors: The Substance Measured Is Often Not in the Record

Within the 825 indexed records, 494 (59.9%) carried no marker descriptor of any kind and only 279 (33.8%) carried at least one specific analyte descriptor (Table 1). In three records out of five, the molecular substance the study set out to measure cannot be recovered from the metadata: the record establishes that the article concerns depression and biomarkers, but not which biomarker.

**Table 1.** **Corpus structure, indexing yield, sampling matrix and compartment recording by publication type**.

Section	Category	n	%
A. Corpus (Tier 1)	Records retrieved	1,166	100
	Indexed in MEDLINE (Tier 2)	825	70.8
	Not indexed (no MeSH of any kind)	341	29.2
B. Decomposition of the unindexed	Structurally excluded (rate from closed year 2024)	≈258	22.1
	Awaiting indexing (residual lag)	≈83	7.1
C. Marker descriptors (within Tier 2, N = 825)	≥1 specific analyte descriptor	279	33.8
	Class-level descriptor only	52	6.3
	No marker descriptor of any kind	494	59.9
D. Sampling matrix (within Tier 2, N = 825)	Peripheral only (/blood, /urine)	159	19.3
	Central only (/CSF)	2	0.2
	Dual-compartment co-indexing (peripheral + central)	6	0.7
	No site qualifier recorded	658	79.8
E. Compartment recording by publication type (within Tier 2)	Primary studies with a compartment recorded	73/188	38.8
	Reviews with a compartment recorded	94/637	14.8

Notes. Percentages are of Tier 1 in section A, of Tier 2 in sections C and D, and of the relevant publication-type stratum in section E. Section B applies the unindexed rate of the closed year 2024 (70/317 = 22.1%) to the full window, assuming a stable structural exclusion rate, and attributes the residual to lag. The association in section E is significant (uncorrected Pearson χ^2^ = 52.1, 1 df, *p* < 0.001; odds ratio (OR) 3.67, 95% confidence interval (CI) 2.54–5.29). CSF, cerebrospinal fluid.

### Sampling Matrix: The Compartment Is Absent From Four Records in Five

A site qualifier was absent in 658 of the 825 records (79.8%), and where present it was almost always peripheral (Table 1). In a random sample of 50 of these unqualified records, only 7 (14.0%) named a sampling matrix anywhere in the title, abstract or substance fields, so for the great majority the compartment is absent from the record altogether rather than merely from the MeSH field. This corpus-wide figure is nonetheless retrieval-dependent and should not be read as a property of the field: it is depressed by the review-dominated composition of the corpus (77.2%), a consequence of the retrieval strategy documented in Methods. The stratified estimates in Section “Compartment Recording Tracks Qualifier Eligibility Rather than Study Design” are the interpretable ones.

### Allowable-Qualifier Eligibility: The Peripheral Compartment Is Unrecordable for an Entire Class of Markers

Interpreting the absent qualifier requires knowing whether it could have been assigned at all. Of the 1211 distinct descriptors appearing in the corpus, 681 (56.2%) do not list /blood among their allowable qualifiers under 2026 MeSH rules; for these descriptors a /blood qualifier could not have been applied under any circumstances, and its absence carries no information about indexing behaviour. A validity check confirmed the partition internally: no descriptor classified as ineligible received a /blood qualifier anywhere in the corpus (0 of 681). That corpus-wide figure is not the informative one, and for the same reason that governs the eligible set below: most of the 681 are population, study-design, disease and drug descriptors, for which a compartment would carry no meaning. Restricted to the 243 marker-level and class-level descriptors, 30 (12.3%) are barred from /blood (**Supplementary Table S1**). The smaller figure is the consequential one, because of what it contains. The class most affected is diagnostically central. CRP, the single most studied inflammatory marker in the corpus (26 records), belongs to the MeSH Blood Proteins category, for which /blood is not an allowable qualifier; the same is true of haptoglobins. These descriptors can, however, receive /cerebrospinal fluid. The vocabulary therefore renders the peripheral compartment structurally unrecordable for a class of plasma proteins while leaving the central compartment recordable for the same descriptors, the exact inverse of where these markers are almost always measured. What reads, in a naïve tabulation, as an indexer’s failure to record that CRP was measured in blood is in fact a rule of the controlled vocabulary: the qualifier does not exist for that descriptor.

This distinction reorganises the marker-level picture (Fig. 2). Among descriptors for which /blood is an allowable qualifier, the canonical blood analytes are in fact qualified at high rates: IL-6 in 15 of 17 records (88%), TNF-α in 8 of 10 (80%), neurofilament proteins in 6 of 6, the opposite of systematic omission. The informative cases are those eligible for /blood that never receive it, where the absence is a true indexing gap rather than a vocabulary constraint. Its extent must be stated carefully: of the 530 /blood-eligible descriptors, 127 (24.0%) receive the qualifier at least once and 403 (76.0%) never do, but that raw figure overstates the omission, for the same reason and in the same direction as the corpus-wide figure above, because the eligible set includes descriptors for which a compartment carries no meaning, such as drug classes (antidepressive agents, 117 records; ketamine, 35), disease terms and study populations. Restricted to class-level and marker-level descriptors, the unqualified cases are microRNAs (0 of 25 records), neurotransmitter agents (0 of 8), omega-3 fatty acids (0 of 10), dopamine (0 of 5), telomerase (0 of 4) and iron (0 of 3). These absences are scattered across marker classes rather than concentrated in one, and unlike the vocabulary constraint documented above they are remediable by indexing practice alone.

**Fig. 2.**
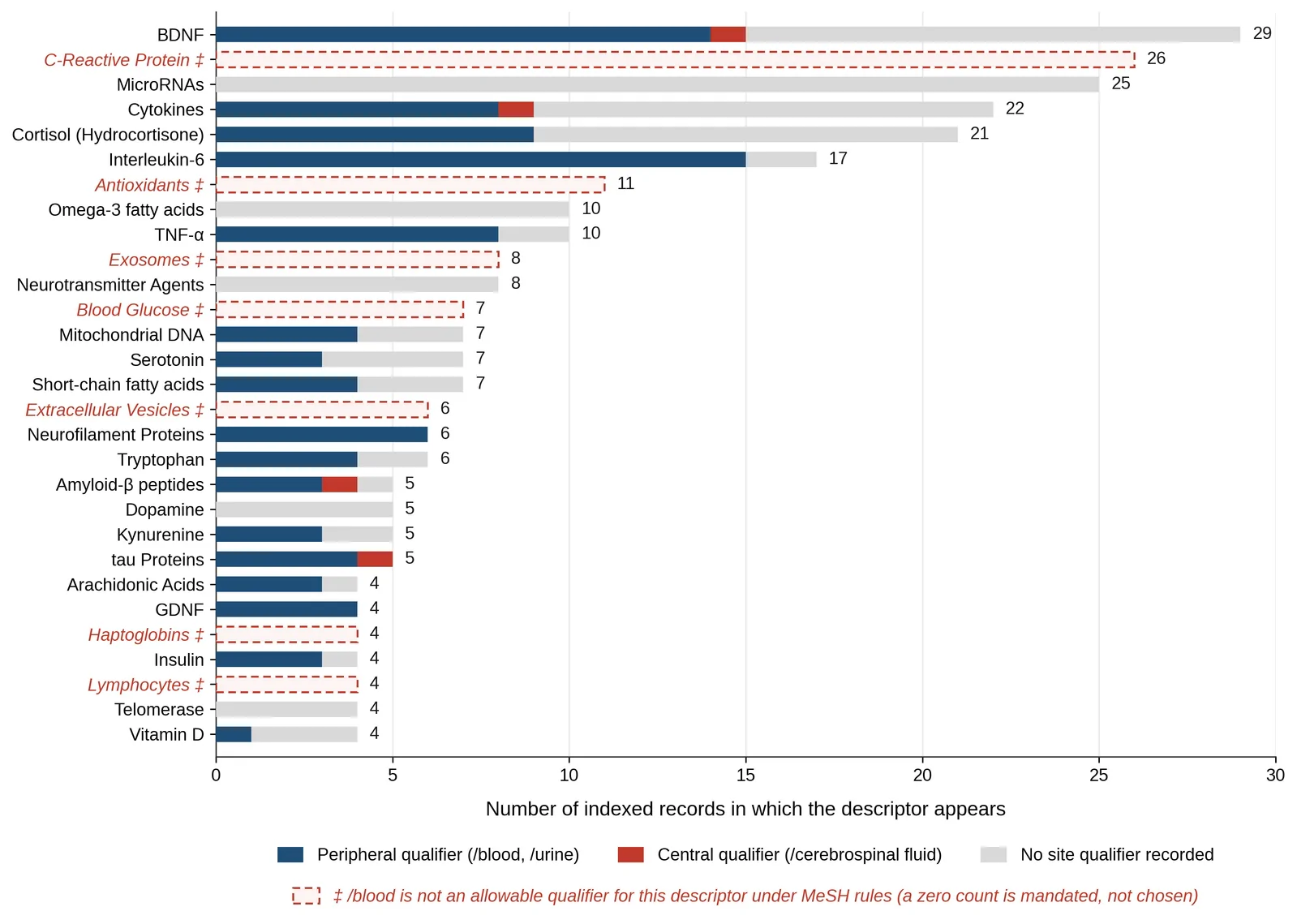
**Is the sampling compartment recorded? Biomarker descriptors by site qualifier and by qualifier eligibility**. Notes. Restricted to marker-level and class-level descriptors appearing in at least four indexed records (n = 29), ordered by record count. Descriptors below that threshold, including iron (0 of 3), are not displayed. Bars marked with a double dagger denote descriptors for which /blood is not an allowable qualifier under MeSH rules, so that a zero count is mandated rather than chosen. Where /blood is allowable, the canonical blood analytes do receive it (interleukin-6, TNF-α, neurofilament proteins, GDNF); the informative exceptions are eligible descriptors that never do (microRNAs, 0 of 25 records; omega-3 fatty acids, 0 of 10; neurotransmitter agents, 0 of 8; dopamine, 0 of 5; telomerase, 0 of 4). Central (/cerebrospinal fluid) qualifiers occur predominantly on neuroaxonal, glial and neurotrophic markers (tau proteins, amyloid-β, neurofilament proteins, GFAP, BDNF), with the remainder attaching to the generic descriptor Biomarkers or to disease terms. BDNF, brain-derived neurotrophic factor; CSF, cerebrospinal fluid; GDNF, glial cell line-derived neurotrophic factor; GFAP, glial fibrillary acidic protein; TNF-α, tumour necrosis factor alpha.

### Granularity: Where a Compartment Is Recorded, It Rarely Identifies the Analyte

Across all 503 /blood occurrences, 349 (69.4%) attached to a descriptor that does not identify the substance measured, and only 154 (30.6%) to a specific analyte (Table 2).

**Table 2.** **Where the sampling compartment is recorded: granularity of the /blood qualifier**.

Descriptor carrying /blood	Type	Occurrences
Biomarkers	Category term	128
Major depressive disorder	Disease	43
Inflammation	Process	22
Depression	Disease	21
Bipolar disorder	Disease	13
Depressive disorder	Disease	11
Other generic and class-level descriptors	—	111
Subtotal — does not identify the substance measured		349 (69.4%)
Interleukin-6	Specific analyte	15
Brain-derived neurotrophic factor	Specific analyte	14
Cortisol (Hydrocortisone)	Specific analyte	9
Tumour necrosis factor-α	Specific analyte	8
Neurofilament proteins	Specific analyte	6
Other specific analytes	—	102
Subtotal — specific analyte		154 (30.6%)
Total /blood occurrences		503

Notes. One occurrence is one record in which the descriptor carries the /blood subheading; a descriptor counts at most once per record. C-reactive protein does not appear in this table because /blood is not an allowable qualifier for it under MeSH rules; its 26 records therefore cannot contribute a /blood occurrence. It is one of fourteen descriptors in the corpus barred from /blood while eligible for /cerebrospinal fluid (**Supplementary Table S8**).

The record more often states that something was measured in blood than what was measured in it. This granularity gap is partly a further consequence of the eligibility constraint documented above: when a specific analyte cannot receive /blood, as with CRP, the qualifier can only attach to a co-indexed generic descriptor that does permit it, such as Biomarkers or the disease term. The mislocation of the qualifier onto generic descriptors is therefore not solely an indexing habit but is in part forced by the structure of the vocabulary itself.

### Compartment Recording Tracks Qualifier Eligibility Rather than Study Design

The corpus is composed predominantly of reviews (637 of 825, 77.2%). Stratified by publication type, primary studies recorded a sampling compartment in 73 of 188 records (38.8%) against 94 of 637 reviews (14.8%), an association unlikely to be due to chance (Table 1). This stratified difference does not, however, identify study design as the dominant determinant. Analyte class was also associated with recording among primary studies (inflammatory markers 72.0% vs 33.7%, *p* = 0.0006; neurotrophic markers 88.9%, *p* = 0.003), but this reflects whether a record carries a specific analyte descriptor rather than which analyte it names: restricted to the 94 primary studies already carrying one, inflammatory markers did not differ from the remainder (*p* = 0.10).

The variable organising both effects is qualifier eligibility. Counting, for each record, how many of its descriptors admit /blood under 2026 MeSH rules yields a monotonic gradient among primary studies: 0%, 5.0%, 16.3%, 30.8% and 59.4% for zero, one, two, three and four or more eligible descriptors (odds ratio [OR] 1.75 per eligible descriptor, *p* < 0.001). In a model including publication type, marker level, indexing depth and eligibility across all 825 records, eligibility remained independently predictive (adjusted OR 1.33, 95% confidence interval [CI] 1.16–1.52) while indexing depth did not (adjusted OR 1.03, 95% CI 0.95–1.11). Considered singly, eligibility and marker level each accounted for approximately twice the variance of publication type (pseudo-R2 0.114 and 0.115 versus 0.056; **Supplementary Material S5b**). Whether the record states where a marker was measured depends less on the study, and less on which marker it names, than on how many opportunities the controlled vocabulary afforded that record to say so.

### The Six Dual-Compartment Records Reflect Topic, Not Design

All six records carrying both a peripheral and a central qualifier are reviews or meta-analyses, as are the two central-only records; none is a primary study. In each of the six, a single descriptor carries both qualifiers, and in five that descriptor is a neuroaxonal, glial or neurotrophic marker (neurofilament proteins, tau proteins, amyloid-β, glial fibrillary acidic protein, brain-derived neurotrophic factor); none concerns an inflammatory marker. The co-occurrence therefore registers that an article discusses both blood and CSF, not that a study collected paired specimens. This is a limitation of what MeSH co-indexing can identify rather than a finding about the conduct of the field.

## Discussion

This analysis set out to ask not whether the recent depression biomarker literature records where its markers were measured, but whether its indexed record is even capable of doing so. The answer is that it is largely not, and for two structural reasons that are independent of any individual indexer’s judgement. The contribution is accordingly of a different order from the biological literature it draws on. That peripheral CRP has limited central specificity, and that blood–CSF coupling is weak and conditional, are established findings and are taken here as premises rather than offered as results. What has not previously been examined, to our knowledge, is whether the indexing infrastructure permits those premises to be tested cumulatively against the literature that generates them. It does not, and the failure is systemic rather than incidental: it originates in the design of the controlled vocabulary and in the composition of the retrievable corpus, not in the conduct of any study or the judgement of any indexer.

The distinction between a measurement of the blood and a measurement of the brain, the distinction on which the translational validity of the entire enterprise depends, is absent from the metadata of four indexed records in five, and where it is present it is usually attached to a descriptor that does not name the marker. Neither failure is principally a matter of indexing diligence. The first is written into the controlled vocabulary; the second follows from the kind of article the field predominantly produces (Fig. 3).

**Fig. 3.**
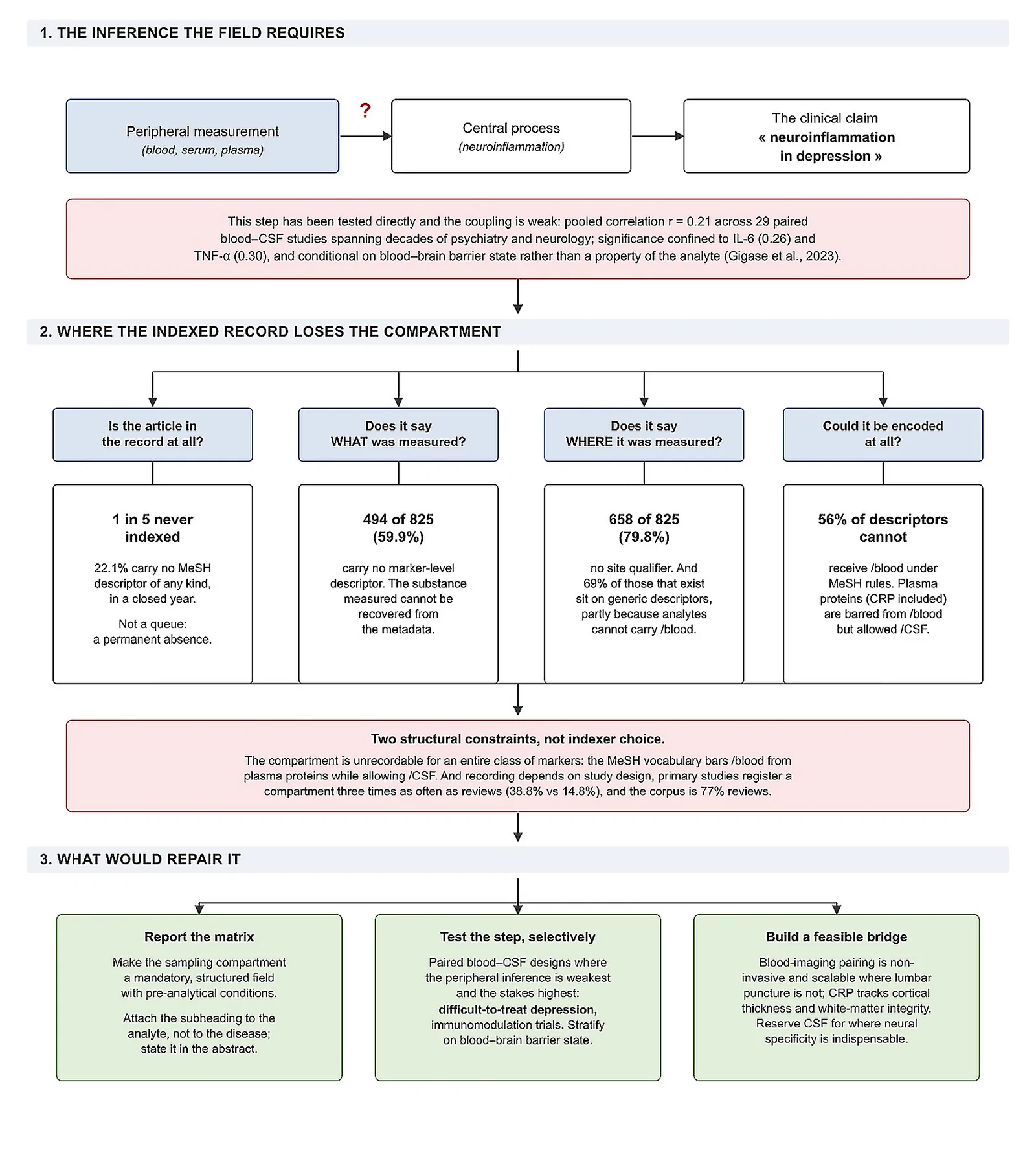
**The inferential chain, the points at which the indexed record loses the sampling compartment, and what would repair it**. Notes. Unless otherwise stated, figures derive from the 825 MEDLINE-indexed records, with denominators given in the Results and Table 1; the unindexed rate in panel 2 is estimated from the closed publication year 2024, and the blood–CSF coupling in panel 1 is from Gigase *et al*. [10]. Two of the four losses in panel 2 are structural rather than behavioural, and the loss at the “where” point is itself partly structural, since analytes barred from /blood displace the qualifier onto generic descriptors. CRP, C-reactive protein; CSF, cerebrospinal fluid; IL-6, interleukin-6; TNF-α, tumour necrosis factor-α.

### What the Record Can and Cannot Encode

The most consequential finding is that the peripheral compartment is not a recordable property of an entire class of markers. Under MeSH rules, 56% of the descriptors in this corpus cannot receive the /blood qualifier at all, and the class barred from it includes the plasma proteins, CRP foremost among them, that constitute the core of the peripheral inflammatory hypothesis in depression. For these descriptors the qualifier does not exist; its absence from a record is not an omission but a structural impossibility. The same descriptors can, revealingly, receive /cerebrospinal fluid, so the vocabulary is not indifferent to compartment in general, it is specifically unable to record the peripheral compartment for the markers most often measured peripherally. This is the inverse of a naïve reading, in which the total absence of /blood on CRP might be taken as evidence that indexers systematically overlook the obvious. They do not: where /blood is an allowable qualifier, the canonical blood analytes receive it at high rates (IL-6 88%, TNF-α 80%, neurofilament proteins 100%). Peripheral BDNF, for instance, shifts measurably with antidepressant treatment [13], and blood neurofilament light chain has emerged as a validated axonal marker across neurological and psychiatric disorders [14, 15]. The apparent silence around markers such as CRP is a property of the vocabulary, not of the indexer, and it means that no amount of improved indexing practice could recover the compartment for these markers without a change to the vocabulary itself. The constraint operates upstream of indexing itself, which bears on a question the National Library of Medicine’s recent transition to algorithmic, assignment, phased in across 2022–2024, would otherwise raise: neither a human indexer nor an automated pipeline can assign a subheading that is not allowable for the descriptor, since allowability is a validation rule applied to any assignment whatever its source. The finding is therefore invariant to the human-versus-algorithmic distinction, and better entity recognition, while it may reduce the genuine omissions we identify among eligible descriptors, cannot restore a qualifier the vocabulary does not provide.

A second and subordinate collapse operates within the peripheral compartment itself. MeSH provides exactly three fluid qualifiers: /blood, /cerebrospinal fluid and /urine, so every peripheral matrix other than urine resolves to the single token /blood. Serum, plasma and whole blood are not separable in the indexed record, although they are not analytically interchangeable: values, protein binding and pre-analytical handling differ across them, and this heterogeneity is itself a recognised source of between-study variance in the peripheral inflammatory literature. Saliva is not represented at the qualifier level at all; it exists only as a descriptor, so a salivary measurement can be recorded as a topic but never as a sampling compartment, and salivary and serum estimates of the same analyte do not correspond [16]. The two constraints compound. Where the compartment survives indexing, it survives at a granularity coarser than the one at which peripheral studies actually differ from one another: the record can at best state that something was measured in blood, never in which blood fraction, and for the non-haematic peripheral matrices it cannot state the compartment at all. A structured sampling-matrix field, as proposed below, would therefore have to be granular rather than binary, since the vocabulary’s single peripheral token is precisely what makes the existing record unable to distinguish comparable from non-comparable measurements.

A second-order consequence follows for retrieval. Because a plasma-protein analyte cannot carry /blood, a search that filters the literature on the peripheral qualifier does not merely miss a few records: it excludes, by construction, the entire class of inflammatory protein markers, returning instead the generic descriptors, Biomarkers, the disease terms, onto which the qualifier is displaced. The peripheral inflammatory literature is, in this precise sense, unfilterable on the very feature that defines it.

### Compartment Recording Tracks What the Vocabulary Permits

The second structural determinant is the composition of the literature itself. Compartment recording is three times as frequent in primary studies as in reviews (38.8% vs 14.8%), and the corpus retrieved by a text-word search for “depression” and “biomarker”, under a publication-type filter admitting three synthesising categories against two primary ones, is more than three-quarters reviews. This is coherent: a primary study collects a specimen and its metadata may register where; a review discusses findings drawn from many compartments and has no single specimen to record. The determinant, however, is not primarily the study design. The stronger and more specific predictor is how many of a record’s descriptors are permitted to carry the peripheral qualifier at all: that gradient is monotonic and survives adjustment, whereas indexing depth does not. What the joint model isolates is therefore not a property of the study, and not a property of the marker, but a property of the vocabulary applied to the record, converging with the descriptor-level constraint of 4.1 and extending it from the descriptor to the record.

This bears directly on the literature most relevant to precision psychiatry. Data-driven biological subtyping of depression, the programme on which stratified treatment depends [4], is itself dominated by synthesising work: in this corpus, articles concerned with subtyping or stratification are proportionally more review-based than the corpus as a whole. Such work records the compartment less not because its authors are less careful, but because it belongs to the stratum in which recording is structurally poorest. The stratification literature thus inherits the blindness of its class: the body of work that aims to resolve depression into biologically distinct subtypes is, at the level of its own metadata, least able to say in which compartment the distinguishing markers were measured.

### The Literature Outside the Record

The records excluded from indexing form a pool with distinctive properties. Of the 70 unindexed records in the closed year 2024, none had acquired a MeSH descriptor when re-checked in August 2026, twenty months after publication, confirming that the exclusion is permanent rather than a lag. All are English-language, and 69 of the 70 carry the PubMed-not-MEDLINE status that marks definitive exclusion. They concentrate in smaller and recently established open-access journals: Frontiers in Psychiatry (6 records), Journal of Clinical Medicine (5), Brain Sciences (5), Cureus (4), with the remainder distributed across 40 further titles. Fifteen name a specific analyte or biological domain in the title, including inflammatory markers, microRNAs, cortisol and gut microbiome measures, and one is a study of monomeric CRP as a biomarker for MDD, a subject squarely within the inflammatory endotype literature and permanently unreachable through the structured record. A structural circularity limits how far this pool can be characterised, and is itself informative. All 70 are review-type articles, and none is a primary study, but this cannot be read as a property of the unindexed literature. The publication types that identify primary research, Clinical Trial and Randomized Controlled Trial, are assigned by indexers during MEDLINE processing; a record that is never indexed can carry only the types supplied by its publisher, in practice Journal Article and, sometimes, Review. Primary studies outside MEDLINE are therefore invisible to a publication-type filter, and do not enter this corpus at all: the same 2024 query without the publication-type restriction returns 401 unindexed records rather than 70. The figure of one article in five reported here is accordingly a conservative estimate confined to review-type articles, and the true extent of structural exclusion is larger. More importantly, the study design that determines whether a sampling compartment is recorded cannot be established for the very records that the structured record omits, because publication type is itself a product of the indexing process. The literature that escapes the record escapes also the categories through which the record could be interrogated.

### Why This Matters Clinically: The Inflammatory Endotype of DTD

These are not merely bibliographic properties. Much current work aims to identify an inflammatory endotype within DTD, directing anti-inflammatory or immunomodulatory strategies to patients in whom a peripheral inflammatory signal reflects a process of clinical consequence [17]. Yet a clinician attempting to assemble that literature from PubMed is defeated at two points. First, CRP, the marker that anchors the endotype, cannot carry the peripheral qualifier, so a compartment-aware search excludes it by construction. Second, the treatment-resistant literature is itself sparsely compartmented: of the 44 records carrying the treatment-resistant descriptor, only 7 record any sampling compartment, and none carries a CRP descriptor at all. The marker most central to the inflammatory hypothesis of treatment-resistant depression is, in this window, absent from the marker-level metadata of that literature entirely.

The clinical cost is not abstract. Trials that enrich on a peripheral inflammatory signal, and stratification schemes that aim to isolate an inflamed subgroup, both depend on finding and pooling the studies that measured it. If those studies cannot be retrieved on the very features that define them, the marker and the compartment, the evidence base for an inflammatory endotype cannot be assembled cumulatively, and each effort restarts from the primary literature by hand.

### What Would Repair It

The corrective is not another candidate marker but a change in what the record keeps, together with the selective testing of the step the record currently obscures. At the level of indexing policy, two changes would address the structural failures directly. First, the allowable-qualifier rules could be revised so that compartment subheadings attach to the specific molecular entity rather than being barred from it; as long as /blood cannot attach to CRP, the compartment will continue to be displaced onto generic descriptors or lost entirely. This is not a departure from MeSH logic but an extension of it: the Entry Combination mechanism already redirects certain analyte–compartment pairs, and the same machinery, applied to plasma proteins, could preserve rather than erase the peripheral compartment. Second, journals could require a structured “sampling matrix” field at submission serum, plasma, whole blood, CSF, saliva, urine feeding directly into the record, so that the compartment does not depend on post hoc indexing of a class the vocabulary cannot express. Such a field would have to be granular rather than binary, since peripheral matrices are not interchangeable: the same analyte measured in saliva and in serum does not yield equivalent estimates [16].

Beyond the record, the underlying validation gap remains and should be addressed where it matters most. Paired blood–CSF designs are costly and are not warranted everywhere, but they are warranted selectively, in precisely the settings where the peripheral inference is weakest and the stakes are highest: DTD and immunomodulation trials, with stratification on blood–brain barrier state (for instance the CSF/serum albumin ratio) rather than the assumption of a fixed peripheral–central coupling. Where lumbar puncture is not feasible, blood–imaging pairing offers a non-invasive and scalable bridge: peripheral CRP, for example, has been related to cortical thickness and white-matter integrity [18, 19, 20], and such designs can test the blood-to-brain step without requiring CSF. What matters is that the bridge be built and, having been built, be recorded in a form the literature can retrieve.

### Optimized PubMed Search Strategies for Depression Biomarker Studies

The findings above imply that no search of the controlled-vocabulary layer can recover the sampling compartment for the markers that matter most. What follows is therefore offered as partial mitigation rather than remedy: recall-benchmarked workarounds whose precision remains unquantified, not validated retrieval protocols. Each was tested for what it recovers of what it should; none was tested for what proportion of what it returns is relevant, a limitation inherent to a design that does not examine full texts. Three approaches follow from the three failure points identified. First, retrieval can be moved from the qualifier layer to the text-word layer, searching the sampling matrix as it is named by authors rather than as it is encoded by indexers. Because MeSH provides a single peripheral token, the terms serum, plasma, whole blood and saliva are recoverable only from title and abstract, and a matrix-term block added to the base query restores a distinction the record itself cannot carry. Second, marker-specific protocols must be asymmetric, because the vocabulary treats the core inflammatory markers differently. IL-6 and TNF-α are eligible for /blood and receive it at high rates, so a qualifier-based search performs well for them. CRP cannot receive /blood under any circumstances, so for this marker, and for the plasma-protein class to which it belongs, only a text-word strategy is available. A single search cannot serve both cases, and a search designed for one silently discards the other. Third, compartment filtering is more efficiently approached through publication type than through the site qualifier, because the compartment is recorded as a function of study design rather than of marker identity. Table 3 sets out the resulting workarounds, benchmarked for recall against the indexed corpus analysed here and executed on 6 August 2026. Three results bear on the argument. First, the text-word strategy reaches 174 records against the 137 that any MeSH-restricted search can reach, of which 37 lie outside MEDLINE entirely and are therefore unreachable by any controlled-vocabulary query however constructed; yet within the indexed corpus it recovers only about half of the records that do carry a compartment (81 of 159 peripheral, 51%), because a substantial fraction of articles do not name the sampling matrix in title or abstract. Its value is one of enrichment rather than completeness: 67% of the indexed records it returns carry a compartment, against 20.2% of the corpus as a whole. Second, the asymmetry between markers is complete rather than gradual. A qualifier-based query recovers 88% of IL-6 records and 80% of TNF-α records, whereas the equivalent query for CRP, “C-Reactive Protein/blood”[MeSH Terms] combined with the depression descriptors, returns no records at all across the entire window. The text-word protocol recovers all 26 CRP records in the corpus, at a cost in precision we have not quantified. The nil return of the control query is not an absence of evidence but a demonstration that the question cannot be posed in the vocabulary: 26 such records exist and none is reachable by the search a reader would naturally construct. Third, restricting to primary studies recovers 70 of the 73 primary records carrying a compartment (96%) and doubles the density of compartment recording relative to the corpus (42% versus 20.2%), while discarding 97 compartment-recorded review records, a trade-off that is acceptable for questions about experimental reporting and unacceptable for questions about the state of a literature. Two limits apply to all four strategies and cannot be designed away. None reaches the records that carry no MeSH descriptor at all, approximately one retrieved article in five, which remain outside any structured search however constructed. And none restores granularity within the peripheral compartment: a text-word block can identify that serum was named in an abstract, but the field cannot be filtered on serum as opposed to plasma, because that distinction has no representation in the record and no consistent representation in author reporting either. No figure reported for these workarounds is an estimate of precision; precision was not assessed, since this would require full-text screening that the meta research design of this study excludes. They should be read as unvalidated workarounds for a defect in the record rather than as a substitute for repairing it. The corrective proposed in Section “What would Repair It”, capturing the sampling matrix at submission as a structured field, would make all of them unnecessary.

**Table 3.** **Retrieval strategies that partially circumvent the vocabulary constraint, with recall against the indexed corpus and residual failure**.

Strategy	Purpose	Records retrieved	Recall against reference set	What it does not recover
A. Text-word matrix block	Bypass the single peripheral qualifier	174 (137 MEDLINE-indexed)	81/159 peripheral records (51%); 89/167 with any compartment (53%)	Half of compartment-recorded records, whose abstracts do not name the matrix; the ∼22% never indexed
B. CRP text-word protocol	Retrieve the marker barred from /blood	154	26/26 CRP records (100%)	Precision unquantified; retrieves the whole depression–CRP literature undifferentiated by compartment
B-neg. CRP/blood MeSH query	Control	0	—	Everything: the query is unformulable
C. IL-6/TNF-α qualifier protocol	Exploit eligibility where it exists	50	15/17 IL-6 (88%); 8/10 TNF-α (80%)	The eligible-but-unqualified records; only 10/26 CRP records, by accident of co-indexing
D. Primary-study filter	Enrich for compartment recording	172 (168 in Tier 2)	70/73 primary studies with a compartment (96%)	97 compartment-recorded review records, excluded by construction

Notes. All searches executed on 6 August 2026; PubMed counts are not exactly reproducible at a distance, since indexing is applied retroactively. Reference sets are those extracted for this study: 159 records with a peripheral qualifier, 167 with any compartment qualifier, 73 primary studies with a compartment, and 26, 17 and 10 records carrying the C-reactive protein, interleukin-6 and TNF-α descriptors. The nil return of the control query B-neg is a property of the allowable-qualifier rules, not of the literature. Recall was benchmarked against the indexed corpus; precision was not assessed (Limitations). Query strings in **Supplementary Material S11**. CRP, C-reactive protein; IL-6, interleukin-6; TNF-α, tumour necrosis factor-α.

## Limitations

Several limitations bound these conclusions.

First, and most fundamental, the analysis is confined to the MeSH Heading field. It does not examine titles, abstracts, author keywords, Substance and Chemical lists or Supplementary Concept Records, any of which may carry compartment or analyte information absent from that field. A marker or compartment we score as unrecorded may therefore be recoverable elsewhere in the record. The frequencies reported here are properties of the controlled-vocabulary layer under our coding scheme, not of PubMed metadata as a whole and still less of the research field; the claim is bounded to the layer through which this literature is filtered, pooled and audited. The magnitude of the shortfall was estimated directly: in a random sample of 50 of the 658 records scored as carrying no site qualifier, 7 (14.0%, 95% CI 7.0–26.2) named a sampling matrix in the title or abstract and none carried it in the Substance or Supplementary Concept fields. The controlled-vocabulary layer therefore understates compartment reporting by roughly one record in seven, and in the remaining 86% the compartment is absent from the record altogether.

Second, the corpus is dominated by reviews (77%), a consequence of the text-word retrieval strategy and the publication-type restriction. It is representative of what a co-occurrence search for “depression” and “biomarker” returns, not of the primary experimental literature, and the low absolute rate of compartment recording partly reflects this. Publication type was treated as an explicit stratifying variable rather than corrected for; the primary-study estimate (38.8%) is the more relevant figure for questions about experimental reporting, and the eligibility gradient of Section “Compartment Recording Tracks Qualifier Eligibility Rather than Study Design”, estimated within that stratum, is less sensitive to corpus composition than either rate.

Third, indexing is a moving target: the 2026 vocabulary and its allowable-qualifier rules were applied to records indexed across 2024–2026, and although no descriptor scored ineligible had received /blood, small vocabulary revisions across years cannot be excluded.

Fourth, the decomposition of the unindexed fraction into structural and lag components assumes the exclusion rate of the closed year 2024 is stable across the window; this is an estimate, not a count.

Fifth, the models comparing determinants of compartment recording are exploratory. They were specified after the descriptive analysis rather than in advance, no correction for multiple comparisons was applied, and some analyte classes were too small for stable estimates: the neuroaxonal and neuroendocrine strata comprised three and twenty-two primary studies respectively and are reported descriptively only. The eligibility gradient is robust across the full corpus, but the class-level comparisons are hypothesis-generating.

Sixth, the eligibility analysis covered /blood and /cerebrospinal fluid, the two qualifiers operationalising the peripheral-central distinction; a fuller vocabulary audit might refine the boundary between mandated and chosen absence.

Seventh, no full-text validation was performed. The sample reported in the first limitation bounds recoverability within the record; it does not establish the correspondence between MeSH compartment coding and the sampling matrix actually reported in the article, which was not measured, and no inter-rater statistic is available. The eligibility analysis establishes that a substantial part of the missing coding could not have been assigned under any circumstances; it does not establish what proportion of the remaining absences corresponds to information present in the full text. That correspondence is the natural next study.

Eighth, the retrieval workarounds of Section “Optimized PubMed Search Strategies for Depression Biomarker Studies” were benchmarked for recall but not for precision: Table 3 establishes what each retrieves of what it should, not what proportion of what it retrieves is relevant. They are presented as workarounds rather than validated protocols; a precision assessment would require the full-text screening excluded by this design.

Ninth, the unindexed pool could be characterised only for the article types admitted by the publication-type restriction. Because Clinical Trial and Randomized Controlled Trial designations are assigned during MEDLINE indexing, primary studies never indexed carry no such designation and fall outside the corpus. The unindexed fraction reported here, and its composition entirely of review-type articles, therefore apply to that subset and not to the unindexed literature as a whole, which the same query without the restriction shows to be substantially larger.

## Conclusions

That peripheral markers correspond poorly to central processes is established. What has not previously been shown is that the bibliographic infrastructure through which this literature is stored and retrieved cannot register the distinction on which that limitation turns. The controlled-vocabulary layer cannot reliably say where a marker was measured, and the failure is structural rather than a remediable lapse of indexing: for an entire class of plasma proteins, CRP among them, /blood is not an allowable qualifier at all, and where it is allowable, whether the compartment is recorded tracks how many of a record’s descriptors may carry the qualifier more closely than it tracks the marker or the study design. No qualifier distinguishes serum, plasma, whole blood or saliva. A distinction that cannot be recorded cannot be aggregated, audited or contested.

The consequence falls on the effort that most needs the record to work. Identifying an inflammatory endotype of difficult-to-treat depression, and stratifying patients accordingly, depends on retrieving exactly the studies the current metadata cannot express. For clinicians, this means the biological matrix must be verified directly in the Methods of primary studies, and an elevated peripheral CRP should not be read as a surrogate of central neuroinflammation, given the weak blood-CSF coupling (r∼0.21). For researchers, retrieval should rest on text-word matrix terms rather than site qualifiers, whose precision remains unquantified, and the sampling matrix should be stated explicitly in the abstract, where it remains retrievable irrespective of indexing. Until the compartment is captured at submission and attached to the analyte rather than to the disease, the peripheral inflammatory literature will remain unsynthesisable on the one variable that determines whether a blood measurement means anything about the brain.

## Availability of Data and Materials

All data supporting the findings of this study are derived from publicly available PubMed/MEDLINE records and from the 2026 MeSH descriptor file (desc2026.xml), both freely accessible from the National Library of Medicine. The search strategies, the parsed record-level dataset, the descriptor classification with the reason for each decision, the allowable-qualifier eligibility table and the analysis scripts are provided as **Supplementary Material (S1–S11)** and permit full reproduction of every count reported. Because PubMed applies indexing retroactively, counts are those of the extraction date stated in the Methods.
