# Supplementary material for: What the Controlled-Vocabulary Layer Cannot Say About Where a Depression Biomarker Was Measured: A Meta Research Analysis of MeSH Sampling-Compartment Coding, January 2024–June 2026
Source: Actas Esp Psiquiatr. 2026 Oct 15;54(5):1732–47. doi: 10.62641/aep.2463 (PMC13714471; doi:10.62641/aep.2463)
Supplement: Supplementary file 1 [file ActEsp-54-5-1732-1747-s1.zip › AEP-2463-Supplementary Material.docx]

**SUPPLEMENTARY MATERIAL**

**What the indexed record cannot say about where a depression biomarker was measured: a metaresearch analysis of MeSH sampling-compartment coding, January 2024 – June 2026**

*Note on organisation. These supplementary sections are arranged to follow the analytical pipeline of the study (retrieval and eligibility → descriptor classification → sampling-matrix coding → record-level analyses → retrieval strategies), which is the order in which the material is generated and reproduced by the attached scripts. This internal order does not coincide with the order of first mention in the manuscript; the two are intentionally distinct.*

**Search strategy and record yield**

Database: PubMed/MEDLINE. Corpus retrieved in July 2026. PubMed counts vary over time, since indexing is applied retroactively and the database continues to acquire records; the retrieval date fixes the corpus. Retrieval was performed at the title/abstract text-word level, not the MeSH level, so that the proportion of self-described biomarker articles carrying a biomarker descriptor remains interpretable rather than circular. A publication-type restriction confined the corpus to article types that report or synthesise empirical findings, excluding editorials, letters, comments and case reports.

Publication-type block (applied to all corpus queries below):

AND (Clinical Trial[Publication Type] OR Randomized Controlled Trial[Publication Type] OR Meta-Analysis[Publication Type] OR Review[Publication Type] OR systematic[sb])

Tier 1 (full window), n = 1,166:

("depression"[Title/Abstract] OR "depressive"[Title/Abstract])

AND ("biomarker"[Title/Abstract] OR "biomarkers"[Title/Abstract])

AND ("2024/01/01"[dp] : "2026/06/30"[dp])

AND [publication-type block]

Tier 2 (MEDLINE-indexed subset), n = 825: ... same ... AND medline[sb]

Lag control (closed year 2024), Tier 1, n = 317:

("depression"[Title/Abstract] OR "depressive"[Title/Abstract])

AND ("biomarker"[Title/Abstract] OR "biomarkers"[Title/Abstract])

AND ("2024/01/01"[dp] : "2024/12/31"[dp])

AND [publication-type block]

Lag control, Tier 2, n = 247: ... same ... AND medline[sb]

Unindexed subset (Table S10), n = 70: ... same as lag control Tier 1 ... NOT medline[sb]

***Note.*** *The PubMed sidebar filter labelled “Systematic Review” corresponds to the subset* systematic[sb] *rather than to Systematic Review[Publication Type], and the two are not equivalent: substituting the publication type for the subset returns 1,172 rather than 1,183 records on re-execution. The subset form is the one applied here.*

**S1. Classification of MeSH descriptors as analyte-level, class-level or non-marker**

Each of the 1,211 distinct MeSH descriptors appearing in the 825 indexed records was assigned to one of three mutually exclusive categories by a deterministic rule applied to the 2026 MeSH descriptor file. A *specific analyte descriptor* (“yes”) is one naming a single measurable molecular species, a defined protein, peptide, hormone, metabolite, lipid or nucleic-acid species or a defined circulating blood-cell count. A *class-level descriptor* names a broad chemical class; the seven such terms are reported separately (Table 2, main text). All remaining descriptors, category terms, generic parent terms, receptors, non-chemical descriptors and administered synthetic drugs, are *non-marker* (“no”).

Because MeSH does not distinguish a substance that is measured from one that is administered: hydrocortisone, vitamin D, iron and other endogenous markers each carry a pharmacologic-action branch, a purely tree-based rule cannot separate the two. The classification therefore applies two explicitly enumerated lists: a synthetic-drug exclusion list and an endogenous-marker retention list (Table S1b). Both are published in full so that every borderline assignment can be inspected and, if contested, revised. The complete descriptor-by-descriptor classification, with the MeSH unique identifier, record frequency and rationale for each term, is provided as Table S1 (attached, *Table_S1_descriptor_classification.tsv*).

Under this rule, **279 of 825 records (33.8%) carry at least one specific analyte descriptor, 52 (6.3%) carry only a class-level descriptor, and 494 (59.9%) carry no marker-level descriptor of any kind**. These figures supersede the provisional counts of the earlier draft; the classification is now reproducible from the attached descriptor table and the record-level dataset (Dataset S4) without any manual step.

**Table S1b. Governed lists used by the classification rule**

Descriptors present in the corpus. The left block is excluded as administered agents; the right block is retained as endogenous markers despite carrying a therapeutic-use branch.

| **Synthetic drug — excluded** | **n** | **Endogenous marker — kept** | **n** |
| --- | --- | --- | --- |
| Ketamine | 35 | Hydrocortisone | 21 |
| Probiotics | 24 | Serotonin | 7 |
| Escitalopram | 6 | Tryptophan | 6 |
| Sertraline | 6 | Dopamine | 5 |
| Prebiotics | 5 | Insulin | 4 |
| Psilocybin | 5 | Vitamin D | 4 |
| Citalopram | 5 | Uric Acid | 3 |
| Curcumin | 4 | Iron | 3 |
| Bupropion | 3 | Melatonin | 3 |
| Levodopa | 2 | Neurosteroids | 2 |
| Fluoxetine | 2 | Glutathione | 1 |
| Dextromethorphan | 1 | Selenium | 1 |
| Celecoxib | 1 | Testosterone | 1 |
| Vortioxetine | 1 | Copper | 1 |
| Nitrous Oxide | 1 | Cholesterol | 1 |
| Lithium Compounds | 1 | Progesterone | 1 |
| Creatine | 1 | Glucocorticoids | 1 |
| Carbidopa | 1 | Calcium | 1 |
| Naltrexone | 1 |  |  |
| Cisplatin | 1 |  |  |
| Catechin | 1 |  |  |
| Minocycline | 1 |  |  |
| Anthocyanins | 1 |  |  |
| Acetylcysteine | 1 |  |  |

*Full membership of both lists (including descriptors not occurring in this corpus) is given in the attached Table S1b.*

**S2. Retrieval and eligibility determination**

The corpus was retrieved with the strategy documented above, (“depression” OR “depressive”)[Title/Abstract] AND (“biomarker” OR “biomarkers”)[Title/Abstract] with the publication-type restriction, yielding 1,166 records (Tier 1). Of these, 825 (70.8%) were indexed in MEDLINE with MeSH descriptors (Tier 2) and constitute the analysable subset; 341 (29.2%) carried no MeSH descriptor of any kind. No eligibility screening was applied within Tier 2: the whole indexed corpus is the denominator.

For every descriptor, allowability of the qualifiers /blood and /cerebrospinal fluid was read directly from the 2026 MeSH descriptor file (allowable-qualifier list per record). Of the 1,211 distinct descriptors, 530 (43.8%) permit /blood and 681 (56.2%) do not; the determination is a property of the controlled vocabulary, not of any record, and is fully reproducible from the attached script (eligibility_table.tsv).

**S3. Site-qualifier coding of the sampling matrix**

Each record was assigned to exactly one sampling-matrix category from its MeSH subheadings: peripheral (/blood or /urine on any descriptor), central (/cerebrospinal fluid), dual-compartment (both present at record level), or none. The categories are mutually exclusive and exhaustive. Peripheral only 159 (19.3%), central only 2 (0.2%), dual-compartment 6 (0.7%), no site qualifier 658 (79.8%).

The vocabulary provides qualifiers for blood, cerebrospinal fluid and urine, but none for saliva; a matrix in routine use for cortisol and other steroid markers is therefore not expressible in the controlled vocabulary at all (see *What the Record Can and Cannot Encode*).

Recoverability outside the MeSH field. A random sample of 50 of the 658 records without a site qualifier (seed 2463) was screened by regular expression for sampling-matrix terms (serum, plasma, whole blood, blood, saliva, cerebrospinal fluid, urine, PBMC, hair, stool) in the title, abstract, Substance (NM) and Registry Number (RN) fields. Seven records (14.0%, 95% CI 7.0-26.2, Wilson) matched in title or abstract; none matched in the substance fields. Matching terms were blood (4) and saliva (3). The controlled-vocabulary layer therefore understates compartment reporting by roughly one record in seven; in the remaining 86% the compartment is absent from the record altogether rather than merely from the MeSH field.

**S4. Record-level dataset and parser**

The record-level classification of all 825 indexed records: PMID, publication type, marker level, sampling matrix, and the descriptors carrying a site qualifier, is provided as Dataset_S4_record_level.tsv (attached). The MEDLINE parser and the descriptor-eligibility script are provided as mesh_eligibility.py. Running the script on the attached PubMed export (pubmed-depression-set.txt) regenerates every count in Table 1 and in Table 2 of the main text. The joint-model script is provided separately as S5b_joint_model.py.

**S5. Compartment recording by publication type**

Primary studies recorded a sampling compartment in 73 of 188 records (38.8%); reviews in 94 of 637 (14.8%). The 2x2 association was tested with a single uncorrected Pearson chi2 test.

chi2 = 52.1, 1 df, p < 0.001; odds ratio 3.67, 95% CI 2.54-5.29. This test was the only inferential procedure in the primary descriptive analysis; the exploratory models reported in S5b are additional, and all remaining outputs are descriptive frequencies. The confidence interval is stated here in the form corrected for consistency with the main text (the earlier draft carried 2.55-5.28 in one location).

**S5b. Determinants of compartment recording: joint model**

Competing determinants of site-qualifier presence were compared in logistic models over all 825 indexed records. Single-predictor pseudo-R2: publication type 0.056; marker level 0.115; indexing depth (n_MeSH) 0.091; number of /blood-eligible descriptors 0.114. Full model (compartment ~ publication type + marker level + indexing depth + eligibility): primary study OR 2.53 (95% CI 1.42-4.51); specific analyte descriptor OR 3.00 (1.93-4.69); indexing depth OR 1.03 (0.95-1.11, p = 0.52); eligible descriptors OR 1.33 (1.16-1.52). Likelihood-ratio tests confirmed that dropping eligibility degraded fit (LR = 17.2, df = 1, p = 3.3e-05) whereas dropping indexing depth did not (LR = 0.42, df = 1, p = 0.52). The publication-type odds ratio is attenuated relative to the unadjusted estimate in S5 (3.67) because eligibility and marker level account for part of that association.

Among primary studies, compartment recording rose monotonically with the number of /blood-eligible descriptors a record carried: 0 of 3, 1 of 20 (5.0%), 7 of 43 (16.3%), 8 of 26 (30.8%) and 57 of 96 (59.4%) for zero, one, two, three and four or more; OR 1.75 per eligible descriptor, p = 1.7e-08, pseudo-R2 0.175.

Analyte class was associated with compartment recording among primary studies (inflammatory markers 72.0% vs 33.7%, Fisher p = 0.0006; neurotrophic markers 88.9% vs 36.3%, p = 0.003). Restricted to the 94 primary studies already carrying a specific analyte descriptor, however, inflammatory markers did not differ from the remainder (p = 0.10): recording tracks whether a record carries an analyte descriptor, not which analyte it names. The neuroaxonal and neuroendocrine strata (three and twenty-two primary studies respectively) are reported descriptively only.

Eligibility was taken from the blood_allowable column of Table S1 and analyte classification follows S1 without modification. All figures above are reproduced by S5b_joint_model.py (attached), run against Dataset_S4_record_level.tsv, Table_S1_descriptor_classification.tsv and pubmed-depression-set.txt; the script verifies the correspondence between the analyte classes and Table S1 at run time and aborts on mismatch.

**S6. Granularity of the /blood qualifier**

Across all 503 /blood occurrences, 349 (69.4%) attached to a descriptor that does not identify the substance measured (category, disease, process or class terms), and 154 (30.6%) attached to a specific analyte. These proportions follow from the analyte classification adopted in Section S1 and supersede the 74.0%/26.0% split of the earlier draft, which used the narrower provisional analyte list. The distribution by descriptor is shown in Figure 2 (main text).

**S7. Per-descriptor site-qualifier distribution (Figure 2 source data)**

Figure 2 plots, for each marker-level and class-level descriptor appearing in at least four indexed records (n = 29), the number of indexed records in which it appears, partitioned by site qualifier. Descriptors for which /blood is not an allowable qualifier are marked; for these the absence of a peripheral qualifier is mandated by the vocabulary and cannot reflect indexing choice. Source counts are contained in the attached descriptor table (Table S1) and blood census (*blood_census.tsv*).

**S8. Vocabulary asymmetry: descriptors barred from /blood but allowed /cerebrospinal fluid**

Fourteen descriptors in the corpus cannot receive /blood under MeSH rules while remaining eligible for /cerebrospinal fluid. For these descriptors the vocabulary permits the central qualifier and bars the peripheral one, although in this corpus neither was in fact assigned: the asymmetry is one of what the vocabulary makes recordable, not of what indexers recorded. The pattern is a property of the category lists, not of any individual descriptor.

| **Descriptor** | **MeSH UI** | **Records** | **/CSF occ.** |
| --- | --- | --- | --- |
| C-Reactive Protein | D002097 | 26 | 0 |
| Haptoglobins | D006242 | 4 | 0 |
| Acute-Phase Proteins | D000209 | 3 | 0 |
| Glycated Hemoglobin | D006442 | 3 | 0 |
| Insulin-Like Growth Factor I | D007334 | 3 | 0 |
| Insulin-Like Peptides | D000096764 | 3 | 0 |
| Somatomedins | D013002 | 2 | 0 |
| Complement System Proteins | D003165 | 1 | 0 |
| Fibrin Fibrinogen Degradation Products | D005338 | 1 | 0 |
| Fibrinogen | D005340 | 1 | 0 |
| Glucose | D005947 | 1 | 0 |
| Insulin-Like Growth Factor II | D007335 | 1 | 0 |
| Oxyhemoglobins | D010108 | 1 | 0 |
| Platelet-Derived Growth Factor | D010982 | 1 | 0 |

**S9. Treatment-resistant depression sub-corpus**

Of the 44 records carrying the descriptor Depressive Disorder, Treatment-Resistant, only 7 record any sampling compartment and none carries a C-reactive protein descriptor. The record-by-record listing is given below and as the attached *Table_S9_treatment_resistant_subcorpus.tsv*.

*Summary: 44 records · 26 reviews, 18 primary · 7 with a compartment (all peripheral) · 0 with a CRP descriptor.*

| **PMID** | **Publication type** | **Sampling matrix** | **CRP descriptor** |
| --- | --- | --- | --- |
| 39414710 | review | none | no |
| 39613748 | review | none | no |
| 38293803 | primary | none | no |
| 40928787 | primary | none | no |
| 37922100 | review | none | no |
| 42036562 | review | none | no |
| 42209433 | review | none | no |
| 41465113 | review | none | no |
| 38570038 | review | none | no |
| 40752781 | review | none | no |
| 40855676 | primary | none | no |
| 38997425 | review | none | no |
| 38326104 | review | none | no |
| 37659714 | review | peripheral | no |
| 39261430 | review | none | no |
| 41437758 | review | none | no |
| 41308888 | primary | peripheral | no |
| 40943622 | review | none | no |
| 40381857 | primary | none | no |
| 40446631 | primary | none | no |
| 38343196 | review | none | no |
| 39674325 | primary | peripheral | no |
| 41577431 | review | none | no |
| 40789196 | review | none | no |
| 42162697 | review | none | no |
| 40419149 | primary | none | no |
| 41171999 | primary | none | no |
| 40081590 | primary | peripheral | no |
| 41155383 | review | none | no |
| 41828665 | review | none | no |
| 41642488 | review | none | no |
| 42411430 | review | none | no |
| 41765237 | review | none | no |
| 39400712 | review | none | no |
| 40012166 | primary | none | no |
| 42032882 | primary | peripheral | no |
| 42015491 | primary | peripheral | no |
| 39929111 | primary | none | no |
| 41109417 | review | none | no |
| 40662502 | primary | peripheral | no |
| 41355453 | review | none | no |
| 42068886 | primary | none | no |
| 38834540 | primary | none | no |
| 41173906 | primary | none | no |

**S10. Bibliographic characterisation of the 70 records that remained unindexed in the closed publication year 2024.**

**A. Article type**

| **Article type** | **n (%)** |
| --- | --- |
| Narrative review | 59 (84.3%) |
| Scoping review | 7 (10.0%) |
| Systematic review | 3 (4.3%) |
| Journal article (type not specified) | 1 (1.4%) |
| Total | 70 (100%) |

**B. Named analyte in title**

| **Title content** | **n (%)** |
| --- | --- |
| No analyte named in title | 55 (78.6%) |
| Analyte or biological domain named in title | 15 (21.4%) |
| Total | 70 (100%) |

**C. Country of first-author affiliation**

| **Country** | **n (%)** |
| --- | --- |
| China | 15 (21.4%) |
| United States | 9 (12.9%) |
| Spain | 7 (10.0%) |
| Brazil | 3 (4.3%) |
| Canada | 3 (4.3%) |
| Germany | 3 (4.3%) |
| India | 3 (4.3%) |
| Italy | 3 (4.3%) |
| Not stated | 3 (4.3%) |
| Romania | 3 (4.3%) |
| United Kingdom | 3 (4.3%) |
| Jordan | 2 (2.9%) |
| Russia | 2 (2.9%) |
| Afghanistan | 1 (1.4%) |
| Australia | 1 (1.4%) |
| Bulgaria | 1 (1.4%) |
| Croatia | 1 (1.4%) |
| France | 1 (1.4%) |
| Indonesia | 1 (1.4%) |
| Iran | 1 (1.4%) |
| Israel | 1 (1.4%) |
| Japan | 1 (1.4%) |
| Poland | 1 (1.4%) |
| Switzerland | 1 (1.4%) |
| Total | 70 (100%) |

**D. Journal**

| **Journal** | **n (%)** |
| --- | --- |
| Frontiers in psychiatry | 6 (8.6%) |
| Brain sciences | 5 (7.1%) |
| Journal of clinical medicine | 5 (7.1%) |
| Cureus | 4 (5.7%) |
| Biomedicines | 3 (4.3%) |
| Consortium psychiatricum | 3 (4.3%) |
| Heliyon | 3 (4.3%) |
| Brain, behavior, & immunity - health | 2 (2.9%) |
| Frontiers in microbiology | 2 (2.9%) |
| Frontiers in neuroscience | 2 (2.9%) |
| International journal of bipolar disorders | 2 (2.9%) |
| Life (Basel, Switzerland) | 2 (2.9%) |
| 31 further journals with one record each | 31 (44.3%) |
| Total (43 distinct journals) | 70 (100%) |

***Notes.*** *Records were retrieved with the corpus query restricted to the closed publication year 2024 and to records outside MEDLINE (NOT medline[sb]), and re-checked in August 2026, twenty months after publication: none had acquired a MeSH descriptor, and 69 of the 70 carry PubMed-not-MEDLINE status indicating definitive exclusion. All 70 are English-language. Article type was taken from the publisher-supplied publication type; because Clinical Trial and Randomized Controlled Trial designations are assigned during MEDLINE indexing, primary studies outside MEDLINE carry no such designation and are not represented here (Limitations). A named analyte was scored from the title only, without full-text assessment and without any judgement of the novelty or maturity of the findings. Country was taken from the affiliation of the first author. Percentages are of 70.*

**S11. Optimized PubMed search strategies (*Optimized PubMed Search Strategies for Depression Biomarker Studies*, Table 3)**

All strategies were executed on 6 August 2026 and benchmarked for recall against the reference sets extracted for this study; precision was not assessed (Limitations). Each query uses the publication-type block defined above, abbreviated below as [PT block].

**Strategy A. Text-word matrix block**

A1, within Tier 2 (n = 137):

("depression"[Title/Abstract] OR "depressive"[Title/Abstract])

AND ("biomarker"[Title/Abstract] OR "biomarkers"[Title/Abstract])

AND ("serum"[Title/Abstract] OR "plasma"[Title/Abstract] OR "whole blood"[Title/Abstract]

OR "saliva"[Title/Abstract] OR "salivary"[Title/Abstract]

OR "cerebrospinal fluid"[Title/Abstract] OR "CSF"[Title/Abstract]

OR "urine"[Title/Abstract] OR "urinary"[Title/Abstract])

AND ("2024/01/01"[dp] : "2026/06/30"[dp])

AND [PT block] AND medline[sb]

A2, within Tier 1 (n = 174):

... same as A1 ... without AND medline[sb]

Recall: 81 of 159 records carrying a peripheral qualifier (51%); 89 of 167 carrying any compartment qualifier (53%). Of the 174 records returned, 37 lie outside MEDLINE and are unreachable by any MeSH-based query.

**Strategy B. C-reactive protein text-word protocol (n = 154)**

("depression"[Title/Abstract] OR "depressive"[Title/Abstract])

AND ("C-reactive protein"[Title/Abstract] OR "CRP"[Title/Abstract]

OR "C-Reactive Protein"[MeSH Terms])

AND ("2024/01/01"[dp] : "2026/06/30"[dp])

AND [PT block] AND medline[sb]

Recall: 26 of 26 records carrying the C-reactive protein descriptor (100%).

**Strategy B-neg. Control query (n = 0)**

"C-Reactive Protein/blood"[MeSH Terms]

AND ("Depression"[MeSH Terms] OR "Depressive Disorder, Major"[MeSH Terms])

AND ("2024/01/01"[dp] : "2026/06/30"[dp])

Returns no records. The descriptor C-Reactive Protein does not admit /blood as an allowable qualifier, so the combination cannot be assigned to any record. The nil return is a property of the allowable-qualifier rules, not of the literature: 26 such records exist in the corpus and none is reachable by this query.

**Strategy C. Interleukin-6 and TNF-α qualifier protocol (n = 50)**

("Interleukin-6/blood"[MeSH Terms] OR "Tumor Necrosis Factor-alpha/blood"[MeSH Terms])

AND ("Depression"[MeSH Terms] OR "Depressive Disorder, Major"[MeSH Terms]

OR "depression"[Title/Abstract] OR "depressive"[Title/Abstract])

AND ("2024/01/01"[dp] : "2026/06/30"[dp])

AND [PT block]

Recall: 15 of 17 interleukin-6 records (88%); 8 of 10 TNF-α records (80%). Incidentally retrieves 10 of the 26 C-reactive protein records through co-indexing, not by design.

**Strategy D. Primary-study filter (n = 172)**

("depression"[Title/Abstract] OR "depressive"[Title/Abstract])

AND ("biomarker"[Title/Abstract] OR "biomarkers"[Title/Abstract])

AND ("2024/01/01"[dp] : "2026/06/30"[dp])

AND (Clinical Trial[Publication Type] OR Randomized Controlled Trial[Publication Type])

AND medline[sb]

Recall: 70 of 73 primary studies carrying a compartment (96%). Of the 168 returned records present in Tier 2, 42% carry a compartment qualifier against 20.2% of the corpus as a whole. Excludes 97 compartment-recorded review records by construction.

**Reference sets**

Derived from Dataset_S4_record_level.tsv and from the descriptor tables: 159 records with a peripheral qualifier, 167 with any compartment qualifier, 73 primary studies with a compartment, and 26, 17 and 10 records carrying the C-reactive protein, interleukin-6 and tumour necrosis factor-α descriptors respectively. The three marker sets were re-derived by descriptor query on 6 August 2026 and intersected with the 825 Tier 2 PMIDs, reproducing the counts reported in the manuscript exactly. PMID lists for every strategy and reference set are provided as Table_S11_strategy_pmids.tsv.

**Attached files**

| **File** | **Contents** |
| --- | --- |
| Table_S1_descriptor_classification.tsv | Full classification of all 1,211 descriptors (analyte / class / non-marker) with UI, frequency and rationale |
| Table_S1b_governed_lists.tsv | Synthetic-drug exclusion list and endogenous-marker retention list |
| Table_S8_vocabulary_asymmetry.tsv | The 14 descriptors barred from /blood but allowed /CSF |
| Table_S9_treatment_resistant_subcorpus.tsv | Record-level listing of the 44 treatment-resistant records |
| Dataset_S4_record_level.tsv | Record-level classification of all 825 indexed records |
| Table_S10_unindexed_2024.tsv | Bibliographic metadata of the 70 records unindexed in the closed year 2024 |
| Table_S11_strategy_pmids.tsv | PMID lists returned by each retrieval strategy and by each reference-set query |
| blood_census.tsv | Per-descriptor count of /blood occurrences (503 total) |
| eligibility_table.tsv | Per-descriptor /blood and /CSF allowability from the 2026 MeSH file |
| mesh_eligibility.py | Parser and eligibility script; regenerates all counts |
| S5b_joint_model.py | Joint logistic-model script; reproduces all figures in Section S5b |
| pubmed-depression-set.txt | MEDLINE export of the 825 indexed records |

***End of Supplementary Material***
